# Patient-Reported Outcomes Among Adults With Congenital Heart Disease in the Congenital Heart Initiative Registry

**DOI:** 10.1001/jamanetworkopen.2024.39629

**Published:** 2024-10-16

**Authors:** Scott Leezer, Rittal Mehta, Anushree Agarwal, Sneha Saraf, Mindi Messmer, Ruth Phillippi, Jamie L. Jackson, Mark Roeder, Aliza Marlin, Noah D. Peyser, Mark J. Pletcher, Richard Krasuski, Matthew Lewis, Leigh Reardon, Arwa Saidi, Ronald Kanter, Satinder Sandhu, Thomas Young, Roni Jacobsen, Emily Ruckdeschel, Adam Lubert, Simran Singh, Ali Zaidi, Dan H. Halpern, Anita Mathews, Thomas Carton, Anitha S. John

**Affiliations:** 1 CURA Strategies, Arlington, Virginia; 2 Division of Pediatric Cardiology, Children’s National Hospital, Washington, DC; 3 Division of Cardiology, University of California San Francisco, San Francisco; 4 Center for Behavioral Health, Nationwide Children’s Hospital, Columbus, Ohio; 5 Adult Congenital Heart Association, Philadelphia, Pennsylvania; 6 Division of Cardiology, Duke University Health System, Durham, North Carolina; 7 Division of Cardiology, Columbia University Irving Medical Center, New York, New York; 8 Division of Pediatric Cardiology, University of California Los Angeles, Los Angeles; 9 Congenital Heart Center, University of Florida, Gainesville; 10 Division of Pediatric Cardiology, Nicklaus Children’s Hospital, Miami, Florida; 11 Division of Pediatric Cardiology, University of Miami, Miami, Florida; 12 Division of Pediatric Cardiology, Ochsner Health, New Orleans, Louisiana; 13 Division of Pediatric Cardiology, Children’s Hospital of Colorado, Denver; 14 Division of Pediatric Cardiology, Children’s Hospital of Philadelphia, Philadelphia, Pennsylvania; 15 Division of Pediatric Cardiology, Cincinnati Children’s Hospital, Cincinnati, Ohio; 16 Division of Cardiology, Weill Cornell Medicine, New York, New York; 17 Division of Cardiology, Mt. Sinai Hospital, New York, New York; 18 Division of Cardiology, NYU Langone Health, New York, New York; 19 Louisiana Public Health Institute, New Orleans

## Abstract

**Importance:**

In the US, there are more than 1.5 million adults living with congenital heart disease (CHD). The Congenital Heart Initiative (CHI) is a digital, online, patient-empowered registry that was created to advance multicenter research and improve clinical care by gathering patient-reported outcomes (PROs) in adults with CHD.

**Objective:**

To report the initial findings of the PROs for adults with CHD from the first 3 years of the CHI.

**Design, Setting, and Participants:**

The CHI was launched nationally on December 7, 2020, as an observational cohort (survey) study. Data were collected virtually through December 31, 2023, and stored on Health Insurance Portability and Accountability Act–compliant cloud-based servers with restricted access. Adults with CHD were recruited through email, social media, general advertising through advocacy organizations, and targeted outreach (telephone and in-clinic recruitment) by clinical centers.

**Main Outcomes and Measures:**

Demographics and validated survey tools on quality of life, mental health, physical activity, and health care utilization were collected at baseline and every 4 months. Descriptive statistics were used to understand the associations between various factors, including the complexity of heart defects, physical activity levels, mental health comorbidities, and socioeconomic and health care access variables. All categorical variables were analyzed using χ^2^ or Fischer exact test as appropriate.

**Results:**

By December 31, 2023, the CHI had enrolled 4558 participants (2530 female [56%]) with a mean (SD) age of 38.5 (13.9) years, representing all 50 states. Approximately 88% of participants (3998 participants) completed at least 1 electronic visit as of December 31, 2023. The most prevalent CHD anatomy included tetralogy of Fallot (883 participants [22%]), transposition of great arteries (452 participants [11%]), and coarctation of the aorta (429 participants [11%]). Approximately 88% of participants (3998 participants) reported at least 1 comorbidity, with arrhythmia (1300 participants [33%]) as the most common cardiac comorbidity and mood disorder (1326 participants [35%]) as the most common noncardiac comorbidity. Among female participants, 45% (1147 participants) reported having had a pregnancy, with 38% (967 participants) resulting in biological children. Participants with complex CHD were less likely than those with moderate CHD to meet recommended physical activity guidelines (χ^2^_2_ = 15.9; n = 3320; *P* < .001), a factor that was more pronounced among female participants. Overall health-related quality of life was rated as good or better by 84% of participants who completed the quality of life PROs (2882 participants), with no difference by CHD complexity.

**Conclusions and Relevance:**

In this cohort study of adults living with CHD, many patients reported mood disorders, but most reported good health-related quality of life. The CHI, the largest registry of adults with CHD, is poised to facilitate multicenter research with the goal of improving clinical outcomes for all adults with CHD.

## Key Points

**Question:**

Can meaningful patient-reported outcomes be obtained through a registry for adults with congenital heart disease (CHD)?

**Findings:**

In this longitudinal cohort study of 4558 participants with CHD enrolled in the Congenital Heart Initiative registry, health-related quality of life was rated as good or better by 84% of participants, with no difference by CHD complexity. Participants with complex CHD were less likely to meet physical activity guidelines.

**Meaning:**

This registry is the largest of adults living with CHD in the US and includes patients with many CHD subtypes, allowing for the meaningful interpretation of patient-reported outcomes in a heterogeneous population of participants.

## Teaser

This cohort study reports the initial findings of patient-reported outcomes for adult patients with congenital heart disease from the first 3 years of the Congenital Heart Initiative registry.

## Introduction

As a result of tremendous advances in diagnostic and surgical techniques, the survival of patients with congenital heart disease (CHD) into adulthood has dramatically improved in the US over the past 50 years.^1,2^ Major barriers, however, have existed in conducting surveillance and long-term outcomes research in this patient population. The lack of a longitudinal registry in the US and the heterogeneity of CHD has resulted in an inability to fully characterize the US population of adults with CHD (ACHD).^3^ In addition, the lack of a robust data collection system in this population hinders research, medical advancements, and the medical community’s understanding of the population’s long-term outcomes.

To address these limitations, a multidisciplinary group of cardiac care practitioners and researchers, registry design experts, patient and family partners, and CHD patient advocacy organizations was assembled to design the Congenital Heart Initiative (CHI), the first ACHD-focused registry in the US. This report highlights the initial findings of patient-reported outcomes (PROs) for patients with ACHD from the first 3 years of the CHI.

## Methods

### Informed Consent

The CHI was reviewed and approved by the Children’s National Hospital institutional review board and was built through the Eureka platform, which has been reviewed and approved by the University of California–San Francisco institutional review board. All participants provide electronic consent. There are no monetary incentives for participation. This cohort study follows the Strengthening the Reporting of Observational Studies in Epidemiology (STROBE) reporting guidelines for observational studies.

### Partner Engagement and Human-Centered Design

Methods for registry design were rooted in a human-centered design approach with the goals of (1) discovering patient needs, (2) designing a platform with patient input, and (3) delivering a registry to specifically address patient needs.^4^ A group of key experts in surveillance research, registry design and management, adult congenital cardiology, quality improvement, and key patient partners and leaders from the Adult Congenital Heart Association (ACHA) was assembled. Plans were established for building the registry with tool selection and testing, website design, and a branding workshop (conducted by CURA Strategies) to establish the vision, mission, and purpose of the registry.^4^

### Registry Tool Selection

The PRO tools were initially selected by the patient, health care practitioner, and researcher community. Baseline surveys included demographics, health services use, and 4 validated tools, including the Quality-of-Life Short Form–20,^5^ Satisfaction with Life Scale,^6^ mental health (Hospital Anxiety and Depressions Scale [HADS]),^7^ and physical activity (International Physical Activity Questionnaire).^8^ Patients specified that electronic visits should not be longer than 10 to 15 minutes.

Sociodemographic and health service use surveys were adapted from the Congenital Heart Survey to Recognize Outcomes, Needs, and Well-Being, in addition to several customized questions designed by the study team.^9^ These questions consisted of a combination of multiple choice and multiple entry responses, as well as open-ended text entry fields. In addition, COVID-19–specific questions were used from the COVID-Citizen Science Study.^10^ CHD anatomy was collected using a multiple-entry check field variable with an open-ended response and then categorized (eTable 1 in Supplement 1) in a hierarchical structure as previously defined.^11^

### Study Design, Setting, and Participants

The CHI is a longitudinal cohort study delivered through a web-based system. Eligibility requirements include (1) being at least 18 years old, (2) having a CHD diagnosis, (3) being able to participate in English, and (4) being able to provide consent and complete surveys independently. Participants are then asked to complete a baseline set of demographic surveys and 4 validated tools. A brief health services update survey and the 4 validated tools are redelivered every 4 months. Race and ethnicity were self-reported by participants as part of the demographic surveys. Data on race and ethnicity are included here to identify potential disparities in health care outcomes and to assess the effectiveness of recruitment strategies in promoting inclusion.

### Eureka Research Platform, Data Collection, and Management

The CHI was hosted on the Eureka Research Platform, which has standardized elements for electronic consent, surveys, and feedback that can be customized for individual studies.^12^ Study data are stored on private, secure, Health Insurance Portability and Accountability Act–compliant, cloud-based servers. Data access is restricted to authorized study personnel.

### Recruitment

CHI recruitment was launched nationally on December 7, 2020. The CHI study was broadly advertised through webinars to the CHD patient community, press releases, social media feeds, and posting on the ACHA website. Participants were also recruited from other Eureka platform studies. To date, there are a total of 155 CHI participants who had participated in Eureka studies before the launch of the CHI. In addition, targeted recruitment was performed at 12 clinical centers through the Congenital Heart Initiative: Redefining Outcomes and Navigation to adult centered care (CHI-RON) study, starting May 2022.

### Registry Governance

The CHI governance is led by a multidisciplinary advisory board of patients with CHD, clinicians, researchers, data scientists, federal funding partners, and patient advocacy organizations. The advisory board is composed of a patient subcommittee and a scientific subcommittee. The patient subcommittee focuses on study recruitment, patient engagement, and research topic prioritization, while the scientific committee oversees project proposals and data analysis plans. As a primary goal of the registry was to facilitate additional research, an intake process was developed for data requests from the scientific community requiring advisory board and executive committee approval before the release of data.

### Statistical Analysis

Data processing, harmonization, and analysis were conducted by an accredited data science team data science team at Children’s National Hospital. Categorical variables collected at baseline and follow-up electronic visits are presented as absolute numbers and percentages. Categorical variables were presented as frequencies and percentages to provide a comprehensive overview. Descriptive statistics were used to understand the associations between various factors, including the complexity of heart defects (classified as simple, moderate, or complex), physical activity levels, mental health comorbidities, and socioeconomic and health care access variables. All categorical variables were analyzed using χ^2^ or Fischer exact test as appropriate, with significance set at 2-tailed *P *< .05. All the assumptions were assessed. All analyses were done using SAS statistical software version 9.4 (SAS Institute). Because this is a descriptive cohort study, the analyses presented represent initial exploratory findings that will serve as a basis for future studies.

## Results

### Registry Demographic Data

**Figure 1.**
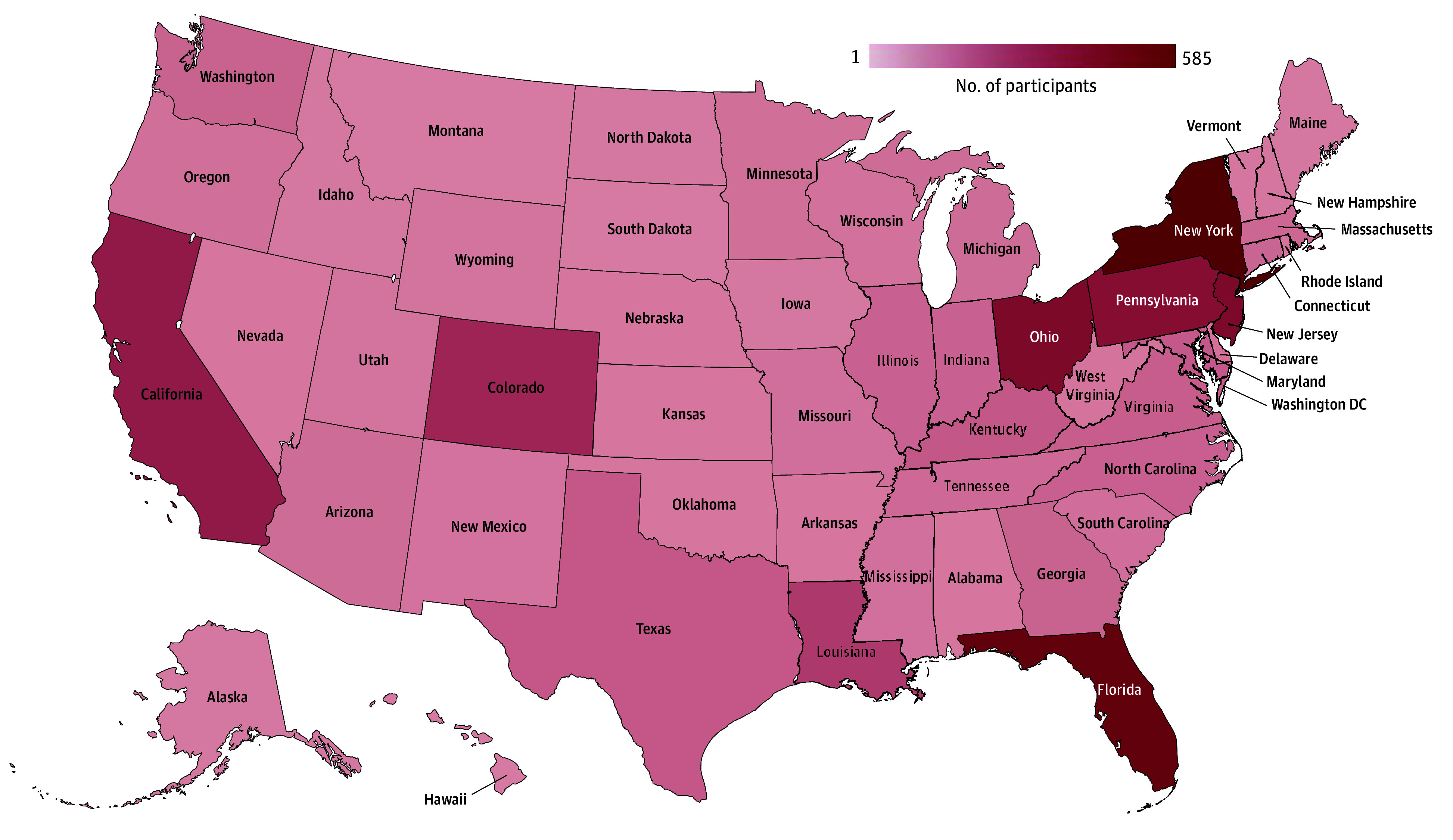
Map of Participants in the Congenital Heart Initiative Figure shows geographic distribution of 4558 participants as of December 31, 2023.

**Figure 2.**
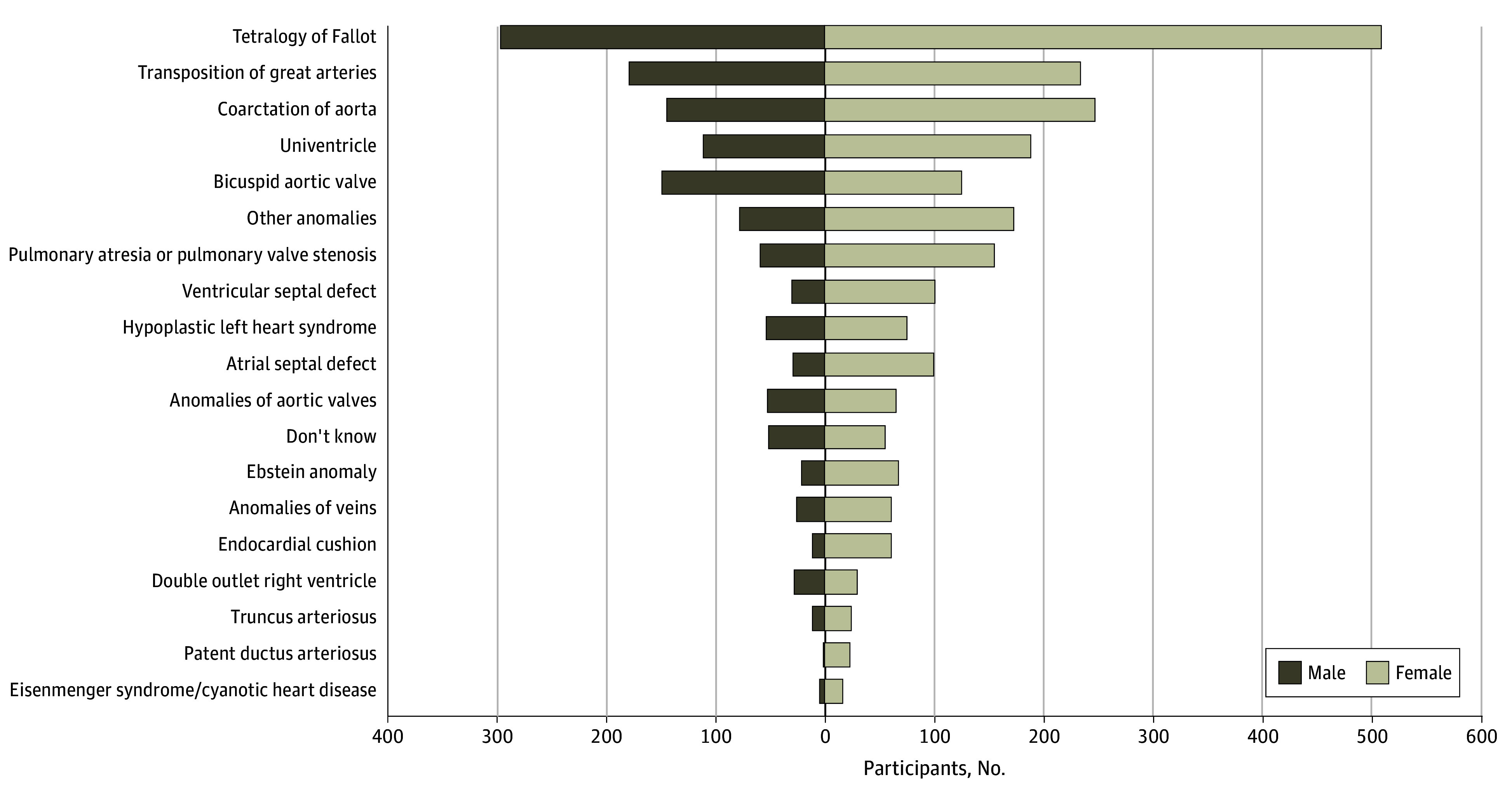
Congenital Heart Disease Subtype by Sex Assigned at Birth Graph shows type of patient-reported congenital heart defect among 3998 Congenital Heart Initiative participants who joined by December 31, 2023, and completed an initial visit.

As of December 31, 2023, a total of 4558 participants (mean [SD] age, 38.5 [13.9]) years; 2530 female [56%]) from all 50 states were enrolled in the CHI (Table and Figure 1); 88% of participants (3998 participants) completed their initial visit, and 62% of participants (2479 participants) completed at least 1 follow-up visit. The participant distribution by sex and patient-reported CHD is shown in Figure 2. The most prevalent CHD anatomy included tetralogy of Fallot (883 participants [22%]), transposition of great arteries (452 participants [11%]), and coarctation of the aorta (429 participants [11%]).

**Table.** Summary of Participant Characteristics Enrolled Through December 31, 2023, by Sex at Birth

Characteristic	Participants, No. (%)				
	All (N = 4558 [100%])	Male (n = 1466 [32%])	Female (n = 2530 [56%])	Other (n = 2 [0.04%])	Unknown (n = 560 [12%])
Age, y					
Mean (SD)	38.5 (13.9)	38.4 (14.7)	38.6 (13.4)	36.5 (23.3)	NA
Median (IQR)	37 (28-47)	36 (28-47)	37 (29-47)	NA	NA
Age group					
18-25	732 (16)	287 (6)	444 (10)	1 (0.02)	NA
26-35	1180 (26)	434 (10)	746 (16)	NA	NA
36-45	991 (22)	346 (8)	645 (14)	NA	NA
46-55	532 (12)	169 (4)	362 (8)	1 (0.02)	NA
56-65	341 (7)	136 (3)	205 (4)	NA	NA
>65	207 (5)	89 (2)	118 (3)	NA	NA
Unknown	575 (12)	5 (0.1)	10 (0.2)	NA	560 (12)
Marital status					
Single	1796 (39)	698 (15)	1097 (24)	1 (0.02)	NA
Divorced	224 (5)	59 (1)	165 (4)	NA	NA
Married	1861 (41)	678 (15)	1182 (26)	1 (0.02)	NA
Other	116 (3)	31 (1)	85 (2)	NA	NA
Unknown	561 (12)	NA	1 (0.02)	NA	560 (12)
Children					
Yes, biological	1517 (33)	549 (12)	967 (21)	1 (0.02)	NA
Yes, adopted	94 (2)	20 (0.4)	74 (2)	NA	NA
No	2306 (51)	874 (19)	1431 (31)	1 (0.02)	NA
Other	81 (2)	23 (1)	58 (1)	NA	NA
Unknown	560 (12)	NA	NA	NA	560 (12)
Race and ethnicity^a^					
Hispanic ethnicity	386 (9)	128 (3)	257 (6)	1 (0.02)	NA
Cuban	51 (1)	16 (0.4)	35 (0.8)	NA	NA
Mexican	92 (2)	27 (0.6)	65 (1)	NA	NA
Puerto Rican	77 (2)	27 (0.6)	50 (1)	NA	NA
Other Hispanic	166 (4)	58 (1)	107 (2)	1 (0.02)	NA
Non-Hispanic	3590 (79)	1330 (29)	2259 (50)	1 (0.02)	NA
Asian	138 (3)	65 (1)	73 (2)	NA	NA
Black or African American	170 (4)	48 (1)	122 (3)	NA	NA
Native American	10 (0.21)	3 (0.06)	7 (0.15)	NA	NA
Pacific Islander	1 (0.02)	NA	1 (0.02)	NA	NA
White	3139 (69)	1166 (26)	1972 (43)	1 (0.02)	NA
White, unknown Hispanic	5 (0.1)	NA	5 (0.1)	NA	NA
Do not know	6 (0.14)	3 (0.07)	3 (0.07)	NA	NA
Multiple races	265 (6)	84 (2)	181 (4)	NA	NA
Other	26 (0.6)	9 (0.2)	17 (0.4)	NA	NA
Unknown	582 (13)	8 (0.18)	14 (0.31)	NA	560 (12)
Education					
Never attended school or only attended kindergarten	1 (0.02)	1 (0.02)	NA	NA	NA
9th to 12th Grade	74 (2)	31 (0.7)	43 (0.9)	NA	NA
High school graduate, General Educational Development, or alternative	427 (9)	173 (4)	253 (6)	1 (0.02)	NA
Some college, no degree	703 (15)	253 (5)	450 (10)	NA	NA
Associate’s degree	308 (7)	89 (2)	219 (5)	NA	NA
Bachelor’s degree	1264 (28)	477 (11)	787 (17)	NA	NA
Graduate or professional degree	1217 (27)	441 (10)	775 (17)	1 (0.02)	NA
Do not know or not sure	4 (0.1)	1 (0.03)	3 (0.07)	NA	NA
Unknown	560 (12)	NA	NA	NA	560 (12)
Annual personal income, $					
0-24 999	780 (17)	282 (6)	497 (11)	1 (0.02)	NA
25 000-49 999	454 (10)	150 (3)	304 (7)	NA	NA
50 000-99 999	738 (16)	300 (6)	438 (10)	NA	NA
100 000-149 999	273 (6)	130 (3)	143 (3)	NA	NA
≥150 000	248 (5)	165 (4)	82 (2)	1 (0.02)	NA
Prefer not to answer	291 (6)	111 (2)	180 (4)	NA	NA
I do not know	107 (2)	26 (0.5)	81 (2)	NA	NA
Unknown	1667 (37)	302 (7)	805 (18)	NA	560 (12)
Annual household income, $					
0-24 999	231 (5)	90 (2)	141 (3)	NA	NA
25 000-49 999	236 (5)	93 (2)	143 (3)	NA	NA
50 000-99 999	608 (13)	224 (5)	383 (8)	1 (0.02)	NA
100 000-149 999	431 (9)	166 (4)	265 (6)	NA	NA
≥150 000	711 (16)	336 (7)	374 (8)	1 (0.02)	NA
Prefer not to answer	340 (8)	134 (3)	206 (5)	NA	NA
I do not know	334 (7)	121 (3)	213 (5)	NA	NA
Unknown	1667 (37)	302 (7)	805 (18)	NA	560 (12)

Abbreviation: NA, not applicable.

^a^ Race and ethnicity were self-identified by the participant; other denotes a category participants could choose if they did not self-identify with the choices offered.

Participants enrolled in the study represented all 50 states, with the highest proportion of patients from New York (463 participants [14%]), Florida (393 participants [12%]), and Ohio (303 participants [9%]). A total of 1183 participants reported their home state in the northeast (35%), 929 were from southern states (28%), 594 were from western states (18%), and 496 were from midwestern states (15%).

In total, 1861 participants (41%) were married and 1692 (37%) had children (Table). Among the participants identifying as female, 1147 (45%) reported having a pregnancy, with 799 of those patients (32%) reporting more than 1 pregnancy; 38% of pregnancies (967 participants) resulted in biological children. The most common diagnoses of women undergoing pregnancy included tetralogy of Fallot (287 women [25%]), shunt lesions (149 women [13%]), and coarctation of the aorta (127 women [11%]), which was reflective of the population of CHI participants.

### PROs Data

**Figure 3.**
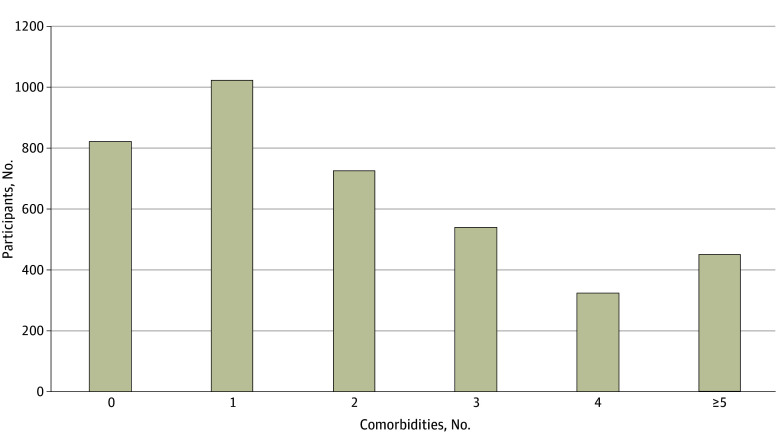
Comorbidity Burden Graph shows number of patient-reported comorbidities per Congenital Heart Initiative participant (n = 3862).

Approximately 12% of participants (560 participants) reported no comorbidities, and 88% (3518 participants) reported 1 or more comorbid conditions (Figure 3 and eTable 2 in Supplement 1). The most common noncardiac comorbidity reported was mood disorders, such as anxiety or depression (1326 participants [35%]), followed by asthma (575 participants [15%]) and anemia (464 participants [12%]). The most common cardiac comorbidity reported was arrhythmia (1300 participants [33%]), followed by hypertension (757 participants [20%]) and heart failure (585 participants [15%]). Of the patients who reported a mood disorder, 24% (318 patients) had normal scores per their HADS reporting, indicating good symptom control. The top 3 most prevalent other chronic health conditions included thyroid issues, migraines, and gastric reflux issues. Most patients had undergone at least 1 cardiac surgery, with only 14% (560 patients) reporting no prior surgical intervention. Seventeen percent of participants (680 patients) reported having had a device such as a pacemaker or a defibrillator implanted; 6% (240 patients) reported having other devices, which included stents, transcatheter prosthetic valves, and implantable recorders.

Most patients reported being currently in care, with 84% (3354 participants) reporting a visit to their cardiologist within the past 2 years and 71% (2841 participants) report visiting a heart doctor in the prior year. Thirty-one percent of patients (1256 participants) reported a gap of 3 years or longer in cardiac care, with the top 5 reasons being that they felt well and did not think they needed to go to a physician (675 participants [54%]), moved (198 participants [16%]), found it too far to drive (141 participants [11.2%]), their parents stopped taking them (162 participants [13%]), or they had changed or lost insurance (183 participants [14.5%]). Fewer patients reported a recent primary care physician (PCP) visit, with 63% (2548 participants) reporting visiting their PCP within 1 year. When asked about reasons for historical gaps in primary care, the top 3 reasons cited were that they felt well (152 participants [36%]), they did not think they needed to see a PCP (116 participants [27.6%]), or they were moving to a different city (81 participants [19.3%]).

**Figure 4.**
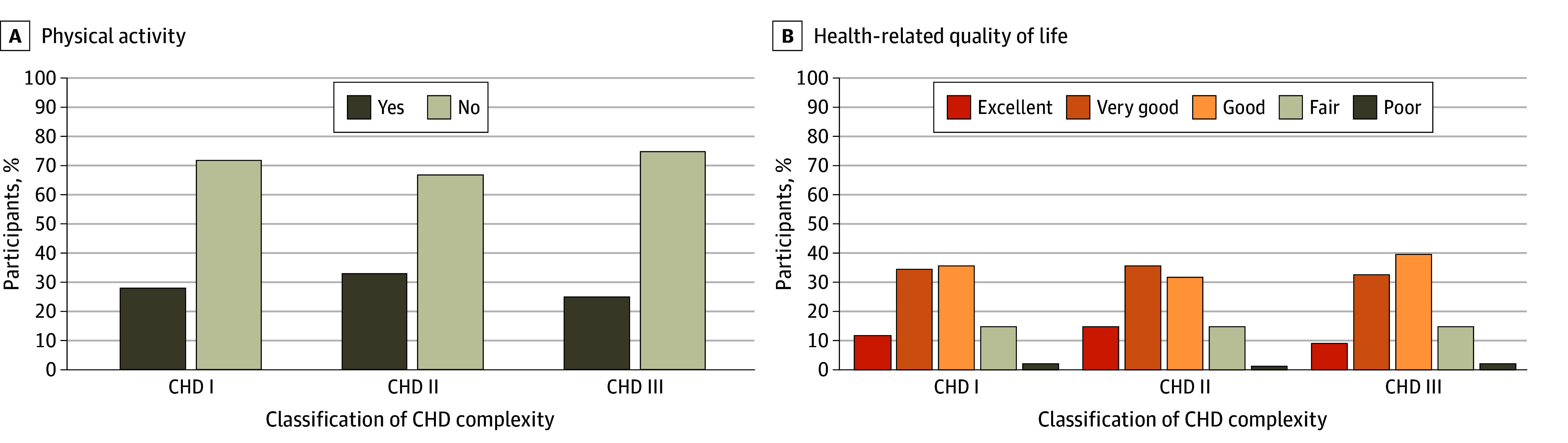
Patient-Reported Outcomes by Congenital Heart Disease (CHD) Complexity Graphs show participant physical activity (A; 3320 participants) and health-related quality of life (B; 3431 participants) by CHD complexity. Physical activity goals are considered met (Yes) if the participant achieves 75 minutes of vigorous activity per week or a combination of vigorous and moderate activity totaling 150 minutes per week.^13^

To assess whether the severity of CHD was associated with exercise levels, participants were stratified by CHD complexity.^11^ Weekly physical activity intervals based on PROs were compared with recommended physical activity guidelines of 75 minutes of vigorous activity per week or a combination of vigorous and moderate activity totaling 150 minutes per week.^13^ Of the 3320 participants with physical activity data, nearly 28% (917 participants) met physical activity guidelines. Initial analysis of baseline data showed that female individuals (228 participants [9%]) were less likely than male individuals (293 participants [20%]) to meet recommended physical activity guidelines. χ^2^ tests of independence were performed, which showed a significant association between CHD severity and the reported level of physical activity (χ^2^_2_ = 15.9; n = 3320; *P* < .001) (Figure 4A). When stratified by CHD anatomy, patients with complex CHD reported lower rates of meeting physical activity recommendations (408 participants [25%]) compared with those with moderate complexity (254 participants [32%]). This was confirmed with the Cochran-Armitage trend test (*P* for trend = .41). Most patients (2882 participants [84%]) reported a good or better health-related quality of life, regardless of CHD complexity (χ^2^ = 0.54; *P* = .46) (Figure 4B).

## Discussion

The overarching goal of the CHI is to improve the quality of life for all adults with CHD while also filling a previously identified gap to advance research, the lack of a longitudinal registry.^3^ In this cohort study, we were able to collect health data from more than 4500 patients from across the US. In addition, our ability to successfully recruit participants demonstrates the feasibility and acceptability of individuals and communities to engage in this digital platform. Initial results show a snapshot of the state of adults with CHD within the US, with the ability to further study physical health and functioning, mental health, and quality of life.

One of the unique aspects of the CHI registry is the strong community engagement. The registry was developed through countless hours of volunteer effort from patients, practitioners, and researchers, resulting in a platform designed by the community it was intended to serve. Human-centered design is a problem-solving qualitative research framework that is grounded in empathy and understanding.^14^ We used this method to discover the needs of the community, design a registry that was customized to patients, and deliver a platform that addressed patient priorities.^4^ Several strategies based on human-centered design methods have been used to promote sustained engagement and participation through sequential surveys, which are often a challenge in digital clinical studies.

Ethical themes and the principles of sound governance for use of Big Data were used to develop the governance structure for the CHI framework.^15,16,17^ As patients consider participation in the CHI, it is important to maintain respect for the individual in the context of seeking answers that apply to and can advance care for all.^18^ Because the data that comprise the CHI are provided by patients, the CHI team understands it is critical to provide the results back to the patient community. Our first and second annual reports can be accessed by the ACHD community on the ACHA website.^19^ To further promote ongoing engagement, data summaries releasing information to the community are included in engagement newsletters and through the ACHA website updates on a periodic basis. In addition, crowdsourcing events to ascertain research priorities are frequently undertaken, often in partnership with the ACHA.

The CHI governance structure is guided by several committees and cores, including a multidisciplinary advisory board composed of numerous partners, including physicians, researchers, and patients. Importantly, patients are part of the governance board and part of the executive leadership of the registry. They provide approval and critiques to investigators proposing research projects. In addition, data summaries from the registries are reviewed by each group and are not released for distribution until both the patient and clinical practitioners review the format and offer clarifications.

One of the major goals of the CHI is to facilitate multicenter research, which plays a pivotal role in advancing understanding of long-term outcomes in ACHD. Increasing sample size, recruiting diverse patient populations, improving reproducibility, and facilitating clinical trials are all key advantages that multicenter research offers in the study of rare diseases. The CHI provides a research ready cohort of patients while also providing key updates on the needs of adults with CHD within the US. There are 2 current multicenter, substudies of the CHI registry: (1) addressing the impact of gaps in recommended care for adults with CHD and (2) establishing how to include adults with CHD with neurodevelopmental disabilities (NDDs) in patient-centered outcomes research.

The first substudy, CHI-RON, is a collaboration between the CHI and PCORnet (the National Patient-Centered Clinical Research Network), an integrated network with health care encounter data from more than 30 million people across the US.^20^ The CHI-RON study is a first-of-its-kind registry for adults with CHD that collects general population data from multiple sources. For the 3027 participants who have been recruited to the CHI-RON study, the option to have 3 distinct types of data included in their profiles—PROs, claims, and electronic health records—was presented at enrollment. The CHI-RON substudy seeks to understand the impact of receiving guideline-directed cardiac care on long-term outcomes in adults with CHD.^11^ A limitation of the CHI data is the reliance on PROs alone. The CHI-RON study allows for linking of different data types, which provides a way to perform concordance studies and identify potential bias.

A second substudy, Achieving Equity: Inclusion of Adults with Congenital Heart Disease Living with Neurodevelopmental Disability in Patient Centered Outcomes Research, aims to improve access for individuals with NDDs and CHD. Unfortunately, a key demographic that remains underrepresented in the CHI is patients with NDD. To be eligible for the CHI, participants must be able to complete the PRO tools offered through the CHI independently. As a result, individuals with CHD and NDD are often ineligible to participate, representing a major gap in PRO research in ACID. To address this gap, we plan to modify our existing successful CHI engagement rubric to create an alternate pathway for inclusion of patients with CHD and NDD to participate in the CHI.

Future directions of the CHI include improving engagement, including partnerships with other CHD-focused registry platforms that are currently lacking in PRO metrics. The CHI is moving toward these goals through (1) using social media to solicit research priorities from the patient community, (2) leveraging existing partnerships for improved data dissemination, and (3) working with interested researchers to use the existing data within the CHI. Our hope is that researchers will use the CHI to conduct future randomized clinical trials and/or comparative effectiveness research capitalizing on the streamlined capacity for data collection and data integration.

### Limitations

One of the major limitations of the registry is that it currently contains PRO data only. Recall bias, underlying neurocognitive challenges, and survey fatigue can all limit continued participation in the CHI. There is also participant bias as individuals concerned about their health issues and those who have access to and are more comfortable with technology are likely overrepresented. There is a higher percentage of female participants, with larger distributions in certain parts of the country. In addition, there is a lack of diversity, with most participants identifying as non-Hispanic White and one-half reporting a college degree. This can limit generalizability to the entire US-based ACHD population. For example, the high prevalence of mood disorders may not seem consistent with the high percentage of patients with good health-related quality of life. This may correspond with the fact that participants with higher socioeconomic status have access to increased resources, including mental health care. This is further supported by the fact that 24% of those reporting mood disorders had normal scores on their HADS PRO, suggesting these patients are adequately treated. To address these issues, a major goal of the CHI-RON substudy is to increase racial and ethnic diversity and geographic distribution of participants. This combined with the use of recurrent, validated PRO tools are strategies being used to mitigate potential bias. Other challenges with registry platform limitations in survey delivery and verification are a common issue among all digital studies.

## Conclusions

In summary, the development of the CHI highlights how digital studies can promote innovations in research and facilitate patient-partner engagement, especially in rare disease conditions. Designed and fueled by patients, the CHI registry serves as a tool to collect PROs while also soliciting the needs of the patient community regarding future research. It also demonstrates the feasibility of the CHI registry as a tool that unites the community of adults with CHD, health care practitioners, and researchers. As the CHI continues to grow and develop, increasing the diversity of participants will be critical to understanding the needs of the ACHD community across the US. Additional partnerships with other organizations, continued innovation in data usage, and improved community engagement will guide future research toward ultimately improving quality of life for all adults with CHD.

## Notes

Supplement 1. **eTable 1.** Summary of CHI Patient-Reported CHD Anatomy and Hierarchical Categorization **eTable 2.** Frequency of Co-Morbid Conditions amongst CHI Participants

Supplement 2.
Data Sharing Statement
