# Supplementary material for: Patient-Reported Outcomes Among Adults With Congenital Heart Disease in the Congenital Heart Initiative Registry
Source: JAMA Netw Open. 2024 Oct 16;7(10):e2439629. doi: 10.1001/jamanetworkopen.2024.39629 (PMC11581669; doi:10.1001/jamanetworkopen.2024.39629)
Supplement: Supplement 1. — eTable 1. Summary of CHI Patient-Reported CHD Anatomy and Hierarchical Categorization eTable 2. Frequency of Co-Morbid Conditions amongst CHI Participants [file jamanetwopen-e2439629-s001.pdf]

Supplemental Online Content

Leezer S, Mehta R, Agarwal A, et al. Patient-reported outcomes among adults with congenital heart disease in the Congenital Heart Initiative registry. *JAMA Netw Open*. 2024;7(10):e2439629. doi:10.1001/jamanetworkopen.2024.39629

eTable 1. Summary of CHI Patient-Reported CHD Anatomy and Hierarchical Categorization

eTable 2. Frequency of Co-Morbid Conditions amongst CHI Participants

This supplemental material has been provided by the authors to give readers additional information about their work.

© 2024 Leezer S et al. *JAMA Network Open.*

eTable 1. Summary of CHI Patient-Reported CHD Anatomy and Hierarchical Categorization

Patient-Reported Congenital Heart Defect	CHI Congenital Heart Disease Hierarchy	ACHD-AP Classification[1]
Eisenmenger syndrome/cyanotic heart disease	1. Eisenmenger syndrome/cyanotic heart disease	III
Hypoplastic left heart syndrome (HLHS)	2. Hypoplastic left heart syndrome (HLHS)	III
Fontan or Single ventricle or Double inlet left ventricle or Tricuspid atresia	3. Univentricle	III
Transposition of great arteries (TGA) or Double outlet right ventricle (DORV)	4. Transposition of great arteries (TGA) or Double outlet right ventricle (DORV)	III
Tetralogy of Fallot (TOF) or Pulmonary atresia (with VSD) and Ventricular septal defect (VSD)	5. Tetralogy of Fallot (TOF)	TOF
Truncus arteriosus	6. Truncus arteriosus	III
Atrial septal defect (ASD)- Pulmonary arterial hypertension (PAH)	7. Atrial septal defect (ASD)- Pulmonary arterial hypertension (PAH)	II
Atrioventricular septal defect	8. Endocardial cushion	II
Anomalies of Aorta	9. Anomalies of Aorta	II
Coarctation of aorta (CoA)	9. Coarctation of aorta (CoA)	II
Ebstein anomaly	10. Ebstein anomaly	II
Pulmonary atresia (PA) (alone) or pulmonary valve stenosis (PV)	11. Anomalies of Pulmonary atresia (PA) or pulmonary valve stenosis (PV)	II
Anomalies of great veins or pulmonary veins (e.g., PAPVR, TAPVR, Scimitar, etc.)	12. Anomalies of veins	II
Subaortic stenosis	13. Subaortic stenosis	II
Patent ductus arteriosus (PDA)	14. Patent ductus arteriosus (PDA)	I
Ventricular septal defect (VSD)	15. Ventricular septal defect (VSD)	I
Aortic valve stenosis or Bicuspid aortic valve	16. Anomalies of aortic valves (AV)	I
Atrial septal defect (ASD)	17. Anomalous coronary artery	I
Other (open-ended text entry)	18. Other anomalies	
Don't know	19. Unknown	

1. Patient-reported CHD anatomy is classified as simple (I), moderate (II), or "great complexity" (III) as defined by the American College of Cardiology and the American Heart Association (Stout, 2019).

© 2024 Leezer S et al. *JAMA Network Open.*

eTable 2: Frequency of Co-Morbid Conditions amongst CHI Participants

Non-Cardiac Morbidities	n (% of Participants)
Mood disorder	1326 (33%)
Asthma	575 (14%)
Anemia	464 (12%)
Obstructive sleep apnea	305 (8%)
Arthritis, gout, lupus	287 (7%)
Diabetes, pre-diabetes or sugar related problem	273 (7%)
Liver disease	266 (7%)
Cancer or malignancy of any kind	158 (4%)
An ulcer in stomach	122 (3%)
Lung disease	117 (3%)
Kidney failure	57 (1%)
Other chronic health problems	572 (14%)
**Cardiac Morbidities**	**n (% of Participants)**
Arrhythmias or heart rhythm problems	1300 (33%)
High blood pressure (hypertension)	757 (19%)
Congestive heart failure	585 (15%)
High cholesterol	418 (11%)
A stroke	245 (6%)
Clotting disoder	116 (3%)
Bleeding disorder	96 (2%)
Heart attack (coronary artery disease or myocardial infarction)	65 (2%)

© 2024 Leezer S et al. *JAMA Network Open.*
