# Supplementary material for: Patient-Reported Outcomes Among Adults With Congenital Heart Disease in the Congenital Heart Initiative Registry
Source: JAMA Netw Open. 2024 Oct 16;7(10):e2439629. doi: 10.1001/jamanetworkopen.2024.39629 (PMC11581669; doi:10.1001/jamanetworkopen.2024.39629)
Supplement: Supplement 2. — Data Sharing Statement [file jamanetwopen-e2439629-s002.pdf]

Data Sharing Statement

Leezer. Patient-Reported Outcomes Among Adults With Congenital Heart Disease in the Congenital Heart Initiative Registry. *JAMA Netw Open*. Published October 16, 2024. doi:10.1001/jamanetworkopen.2024.39629

Data
Data available: No

Additional Information
Explanation for why data not available: Data will be made available on request through proposal development form. Request goes through review by patients and researchers prior to approval.
